# ADA2 genotype and enzyme activity may predict vasculitic or hematologic DADA2 phenotype

**DOI:** 10.70962/jhi.20250108

**Published:** 2026-06-16

**Authors:** Philipp Peters, Johanna Schepp, Michael H. Albert, Horst von Bernuth, Norbert Blank, Jürgen Brunner, Gregor Dückers, Lisa Ehlers, Susan Farmand, Dirk Föll, Elisabeth Förster-Waldl, Janina Gburek-Augustat, Sujal Ghosh, Lisa Göschl, Marc Hömberg, Manfred Hönig, Tilmann Kallinich, Christian Klemann, Leah Klingel, Udo Kontny, Lisa-Maria Kuhn, Peter Lamprecht, Kai Lehmberg, Almut Meyer-Bahlburg, Ingo Müller, Michaela Nathrath, Tim Niehues, Prasad T. Oommen, Jana Pachlopnik Schmid, Seraina Prader, Alexander Puzik, Raffaele Renella, Ansgar Schulz, Andrea Skrabl-Baumgartner, Sven Starke, Teresa K. Tarrant, Michael Hershfield, Bodo Grimbacher, Fabian Hauck

**Affiliations:** 1 Department of Pediatrics, Dr. von Hauner Children’s Hospital, University Hospital, Ludwig-Maximilians-Universität München, Munich, Germany; 2 Department of Pediatrics and Adolescent Medicine, University Medical Center, University Ulm, Ulm, Germany; 3 Department of Pediatric Respiratory Medicine, Immunology and Critical Care Medicine, Charité University Medicine, Berlin, Corporate Member of Free University and Humboldt University and Berlin Institute of Health, Berlin, Germany; 4 Department of Immunology, Labor Berlin Charité-Vivantes, Berlin, Germany; 5 Charité - Universitätsmedizin Berlin, Corporate Member of Freie Universität Berlin, Humboldt-Universität zu Berlin, and Berlin Institute of Health, Berlin-Brandenburg Center for Regenerative Therapies, Berlin, Germany; 6 German Center for Child and Adolescent Health, Berlin, Germany; 7 Department of Hematology, Oncology and Rheumatology, Internal Medicine V, University Hospital Heidelberg, Heidelberg, Germany; 8 Department of Pediatrics, Medical University Innsbruck, Innsbruck, Austria; 9 Faculty of Medicine and Dentistry, Danube Private University, Krems, Austria; 10 Kinderarztpraxis Am Venner Markt, Mönchengladbach, Germany; 11 German Rheumatology Research Center Berlin - A Leibniz Institute, Berlin, Germany; 12 Division of Pediatric Stem Cell Transplantation and Immunology, Department of Pediatric Hematology and Oncology, University Medical Center Hamburg, Hamburg, Germany; 13 Department of Pediatric Rheumatology and Immunology, University of Muenster, Münster, Germany; 14 Division of Neonatology, Department of Pediatrics and Adolescent Medicine, Intensive Care and Neuropediatrics, Center for Congenital Immunodeficiencies, Medical University of Vienna, Vienna, Austria; 15 Comprehensive Center for Inflammation and Immunity, Medical University of Vienna, Vienna, Austria; 16 Division of Neuropaediatrics, Hospital for Children and Adolescents, University Hospital Leipzig, Leipzig, Germany; 17 Department for Pediatric Oncology, Hematology and Clinical Immunology, Medical Faculty, Center of Child and Adolescent Health, Heinrich Heine University, Düsseldorf, Germany; 18 Division of Rheumatology, Department of Internal Medicine III, Medical University of Vienna, Vienna, Austria; 19 Department of Pediatric Oncology and Hematology, University Children’s Hospital of Cologne, Medical Faculty, University of Cologne, Cologne, Germany; 20 Department of Pediatric Immunology, Rheumatology and Infectiology, Hospital for Childrens and Adolescents, University of Leipzig, Leipzig, Germany; 21 Department of Pediatrics and Adolescent Medicine, University Medicine Greifswald, Greifswald, Germany; 22 Division of Pediatric Hematology, Oncology and Stem Cell Transplantation, University Medical Center, Aachen, Germany; 23 Department of Rheumatology and Clinical Immunology, University of Lübeck, Lübeck, Germany; 24 Department of Pediatric Hemato-Oncology, Psychosomatics and Systemic Diseases, Klinikum Kassel, Kassel, Germany; 25 Children’s Cancer Research Centre and Department of Pediatrics, Klinikum Rechts der Isar, Technische Universität München, Munich, Germany; 26 Centre for Child and Adolescent Health, Helios Klinikum Krefeld, Affiliated with Rheinisch-Westfälische Technische Hochschule University Aachen, Aachen, Germany; 27 Division of Immunology and Children’s Research Center, University Children’s Hospital Zurich, and Pediatric Immunology, Faculty of Medicine, University of Zurich, Zurich, Switzerland; 28 Department of Pediatric Hematology, Oncology and Stem Cell Transplantation, Children’s Hospital, Medical Center – University of Freiburg, Faculty of Medicine, University of Freiburg, Freiburg, Germany; 29 Division of Pediatrics, Pediatric Hematology-Oncology Unit, University Hospital of Lausanne, Lausanne, Switzerland; 30 Department of Pediatrics and Adolescent Medicine, Medical University Graz, Graz, Austria; 31 Department of Pediatric Oncology, Hematology and Hemostaseology, University Hospital Leipzig, University of Leipzig, Leipzig, Germany; 32 Division of Rheumatology and Immunology, Department of Medicine, Duke University School of Medicine, Durham, NC, USA; 33 Institute for Immunodeficiency, Center for Chronic Immunodeficiency, Medical Center, Faculty of Medicine, Albert-Ludwigs-University of Freiburg, Freiburg, Germany; 34 Clinic of Rheumatology and Clinical Immunology, Center for Chronic ImmunodefiVical Center, Faculty of Medicine, Albert-Ludwigs-University of Freiburg, Freiburg, Germany; 35 German Center for Infection Research, Satellite Center Freiburg, Freiburg, Germany; 36 Centre for Integrative Biological Signalling Studies, Albert-Ludwigs University, Freiburg, Germany; 37 RESIST – Cluster of Excellence 2155 to Hanover Medical School, Satellite Center Freiburg, Freiburg, Germany

## Abstract

Deficiency of adenosine deaminase 2 (DADA2) is an autoinflammatory disease with diverse phenotypes. We describe the genetics, phenotypes, and treatment of *n* = 48 DADA2 patients from Germany, Austria, and Switzerland. We report a high incidence of hematological (83%) and immunological features (85%), a comparatively low anti-tumor necrosis factor full-response rate (58%), and a high decision probability (21%) for hematopoietic cell transplantation (HCT). We establish a correlation between genetic variant ADA2 activity and patient ADA2 activity. Remarkably, lower patient ADA2 activity is predictive of neutropenia and shows a trend toward HCT decision, whereas higher patient ADA2 activity is predictive of vasculitis symptoms. Genetic variants with low residual ADA2 activity are significantly more common among patients receiving HCT. Our study corroborates previous observations connecting ADA2 activity and clinical phenotype, which up to now have been mainly based on in vitro data.

## Teaser

Peters et al. describe genetics and phenotypes of *n* = 48 DADA2 patients from Germany, Austria, and Switzerland. The data support the hypothesis that patients with low residual ADA2 activity are prone to bone marrow failure, while high residual ADA2 activity correlates with vasculitis.

## Introduction

Deficiency of adenosine deaminase 2 (DADA2) is an autoinflammatory disease (1) with an estimated prevalence of ∼1 in 222,000 individuals (2). DADA2 was first described in 2014, when two independent groups found that homozygous or compound heterozygous mutations in *ADA2* were disease-causing for a syndrome including polyarteritis nodosa (PAN)–like vasculitis, intermittent fevers, early-onset lacunar strokes, and other neurovascular manifestations (3, 4).

Genetically, all reported disease-causing variants in *ADA2* were homozygous or compound heterozygous and affected all domains of the protein. Functionally, they all led to severely decreased plasma ADA2 activity (3, 4).

ADA2 is primarily expressed by myeloid cells (5, 6, 7) and catalyzes the deamination of adenosine and 2′-deoxyadenosine to inosine and 2′-deoxyinosine (6, 8, 9). Many mechanisms have been proposed to trigger autoinflammation in DADA2 (10, 11, 12); however, the full pathophysiology still remains to be determined.

The clinical picture of DADA2 is heterogeneous; next to vasculitis symptoms and strokes, it also includes immunological (56%) and hematological features (40%) (10). Benign lymphoproliferation was reported in ∼30% of DADA2 patients (10), whereas malignancy is very rare. A genotype–phenotype correlation was described in 2020, when Lee et al. found that patients with a predominantly vasculitis phenotype mostly had missense mutations with at least 3% residual variant activity as measured in vitro, whereas phenotypes of pure red cell aplasia or bone marrow failure were associated with missense mutations with minimal residual variant activity <3% or nonsense variants and insertions/deletions resulting in complete loss of function (13).

Tumor necrosis factor (TNF) inhibitors are considered the gold-standard treatment for the vasculitis phenotype of DADA2. Hematopoietic cell transplantation (HCT) is a valid alternative, especially for patients not responding to TNF inhibitors, with resolution not only of the hematological phenotype but also of the systemic inflammation (14). Furthermore, promising experimental results aiming at an autologous hematopoietic stem/progenitor cell gene therapy have been published (15, 16).

Here, we report a large cohort of 48 DADA2 patients from Germany, Austria, and Switzerland, evaluating the genetics, ADA2 activity, phenotypes, demographics, and treatment modalities, as well as outcomes. Specifically, we provide data on the efficacy of TNF inhibitors and the outcome of HCT and correlate them with patient ADA2 activity. To our knowledge, this is the first study that associates patient ADA2 activity with clinical phenotypes and treatment outcomes of a large age-mixed DADA2 cohort.

## Results

### Reported datasets

A total of 126 responses were collected via the survey tool, but 73 datasets were incomplete. Of the remaining 53 datasets, 5 were duplicates, leaving a total of 48 complete individual patient datasets.

### The DADA2 cohort comprises 48 demographically diverse patients

In the study, *n* = 48 DADA2 patients were enrolled, of which each *n* = 24 (50%) were male or female. Age of DADA2 onset was widespread with a median of 4.0 years (range 1 mo–43 years), with symptoms starting before the age of 18 years in all but *n* = 2 (4%) patients. Age at diagnosis varied accordingly with a median of 15.0 years (range 0.5–50 years), with a median diagnostic delay of 7.8 years (range 0–42 years). Diagnosis was made postmortem in *n* = 1 patient. Median age at last follow up was 17.5 years (range 3–57 years). At the time of analysis, *n* = 3 (6%) patients were deceased: *n* = 1 (2%) either from catheter-associated sepsis, subarachnoid hemorrhage, or without reported cause of death, respectively. The total follow-up after DADA2 diagnosis was 143.8 patient-years. Many patients were treated by different sub-specialties, including *n* = 21 (44%) by immunologists, *n* = 17 (35%) by rheumatologists, and *n* = 7 (15%) by hematologists/oncologists. For a summary of the epidemiological data and baseline information on the cohort, please also refer to Table 1.

**Table 1.** Baseline characteristics

​	*n* ^a^
Female	24 (50%)
Deceased	3 (6.3%)
Hematopoietic stem cell transplantation	10 (21%)
TNF inhibitor treatment	37 (77%)
Biallelic ADA2 variant	46 (96%)
ADA2 activity available	35 (73%)
​	**Median (range)** ^b^
Age at onset [years]	4.0 (0.08–43)
Age at diagnosis [years]	15 (0.5–50)
Age at last follow-up [years]	17.5 (3–57)
Diagnostic delay [years]	7.8 (0–42)
ADA2 activity [mU/g]	2.0 (0–19)
ADA2 activity [mU/ml]	0.56 (0.24–0.68)

a Baseline data given as number (percent) of patients.

b Baseline information given as median (range) on age, diagnosis, and ADA2 activity.

### ADA2 deficiency arises from a wide spectrum of variants that consistently reduce enzyme activity

**Figure 1.**
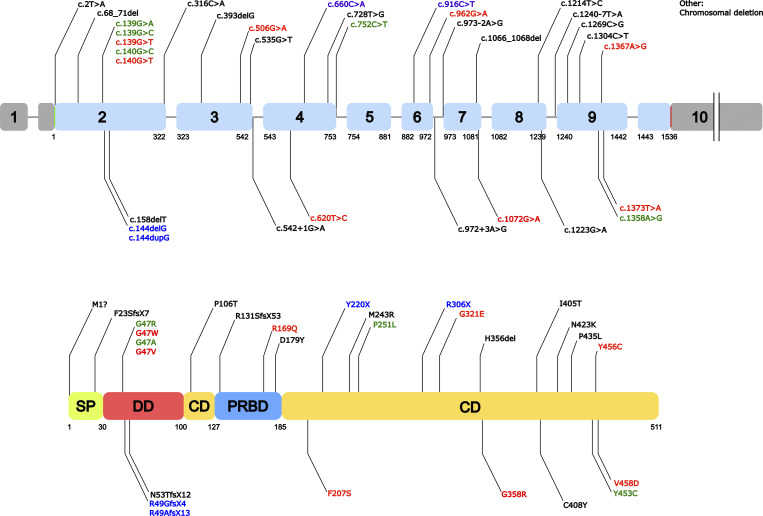
**ADA2 variants are distributed throughout the entire gene.** ADA2 gene (above) and protein (below). Exons 1–10 are depicted as bars, and introns as lines (not to scale). Variants in this cohort are colored in green, red, or blue, depending on residual in vitro activity, as described by Lee et al. (13), corresponding to high (>3% of wild-type activity), low (<3% of wild-type activity), or no (0% of wild-type activity) residual enzyme activity. If the given variant was not described by Lee et al. (13), it was colored black. The protein comprises a signal peptide (SP), dimerization domain (DD), catalytic domain (CD), and putative receptor-binding domain (PRBD).

Biallelic *ADA2* variants could be detected in compound heterozygous (*n* = 27; 56%) or homozygous (*n* = 19; 40%) states in *n* = 46 (96%) patients. In *n* = 1 (2%) patient, only a monoallelic *ADA2* variant could be detected, and in another *n* = 1 (2%), no *ADA2* variant could be found. Both of them, however, showed very low or absent ADA2 plasma activity and were therefore included in the study. 33 distinct ADA2 variants were detected: 20 were missense, two stop gained, one start lost, four splice site, and six insertions/deletions, of which one was in frame (Fig. 1 and Table S1). ADA2 plasma activity was reported in *n* = 35 (73%) of the DADA2 patients and was pathologically reduced in all of them. In 31 of these cases, ADA2 activity was measured in mU/g. The median ADA2 activity for these 31 patients was 2.0 mU/g (range 0–19.0), compared with 130 ± 53 mU/g (mean ± SD) for controls. In 4 DADA2 patients, plasma ADA2 activity was reported as mU/ml and ranged from 0.24–0.68 mU/ml. However, reference values were not provided for these four patients.

### DADA2 manifests with a broad spectrum of vascular, immune dysregulatory, infectious, and hematological features

**Figure S1.**
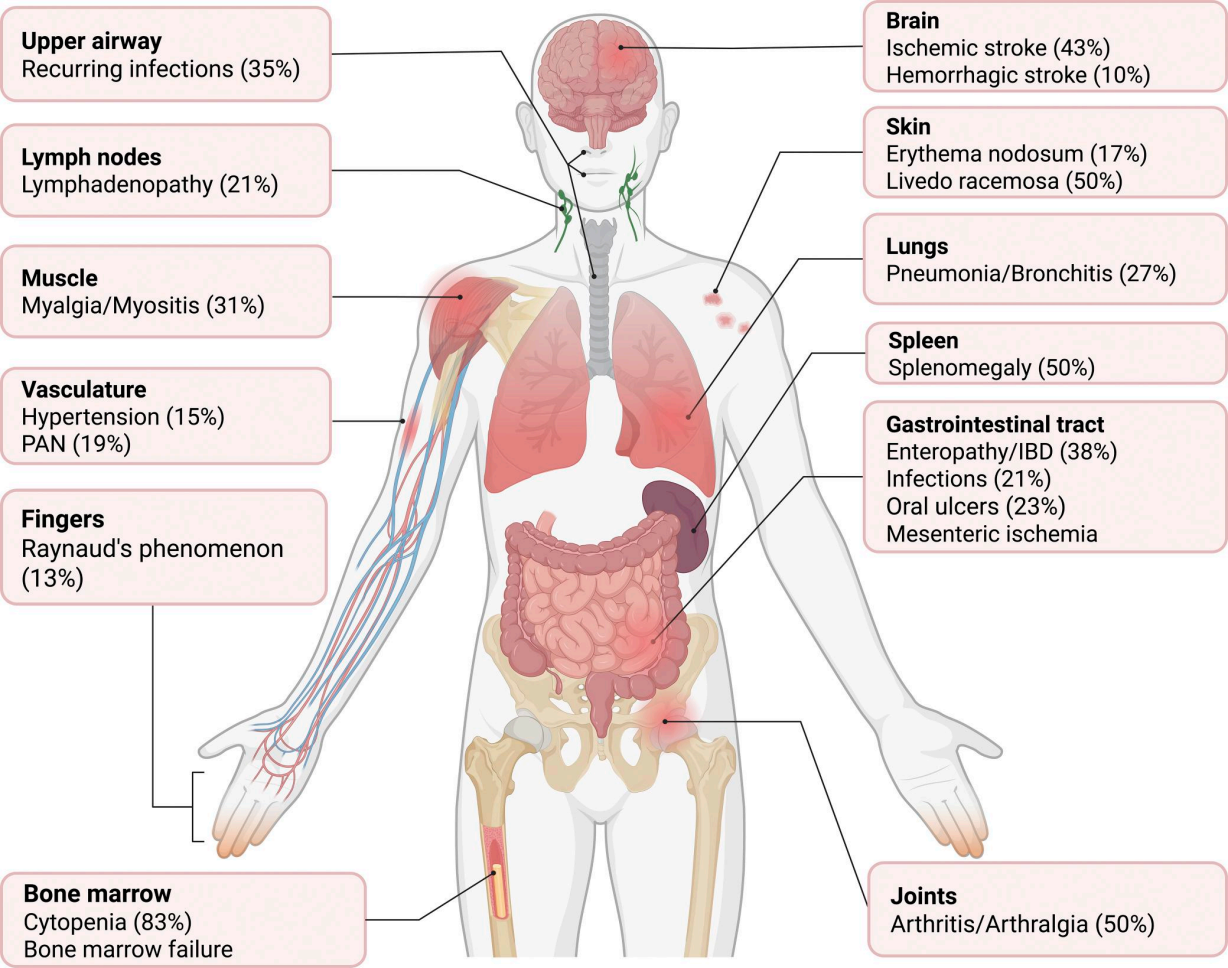
**Common clinical findings in patients with deficiency of ADA2.** Observed frequencies in this cohort shown in parentheses.

Clinical findings reported in the DADA2 cohort mainly comprised vascular manifestations (*n* = 34, 71%), immune dysregulation, including lymphoproliferation (*n* = 41, 85%), hematological alterations (*n* = 40, 83%), immunodeficiency (*n* = 40, 83%), and infections (*n* = 28, 58%) (Fig. S1).

**Figure 2.**
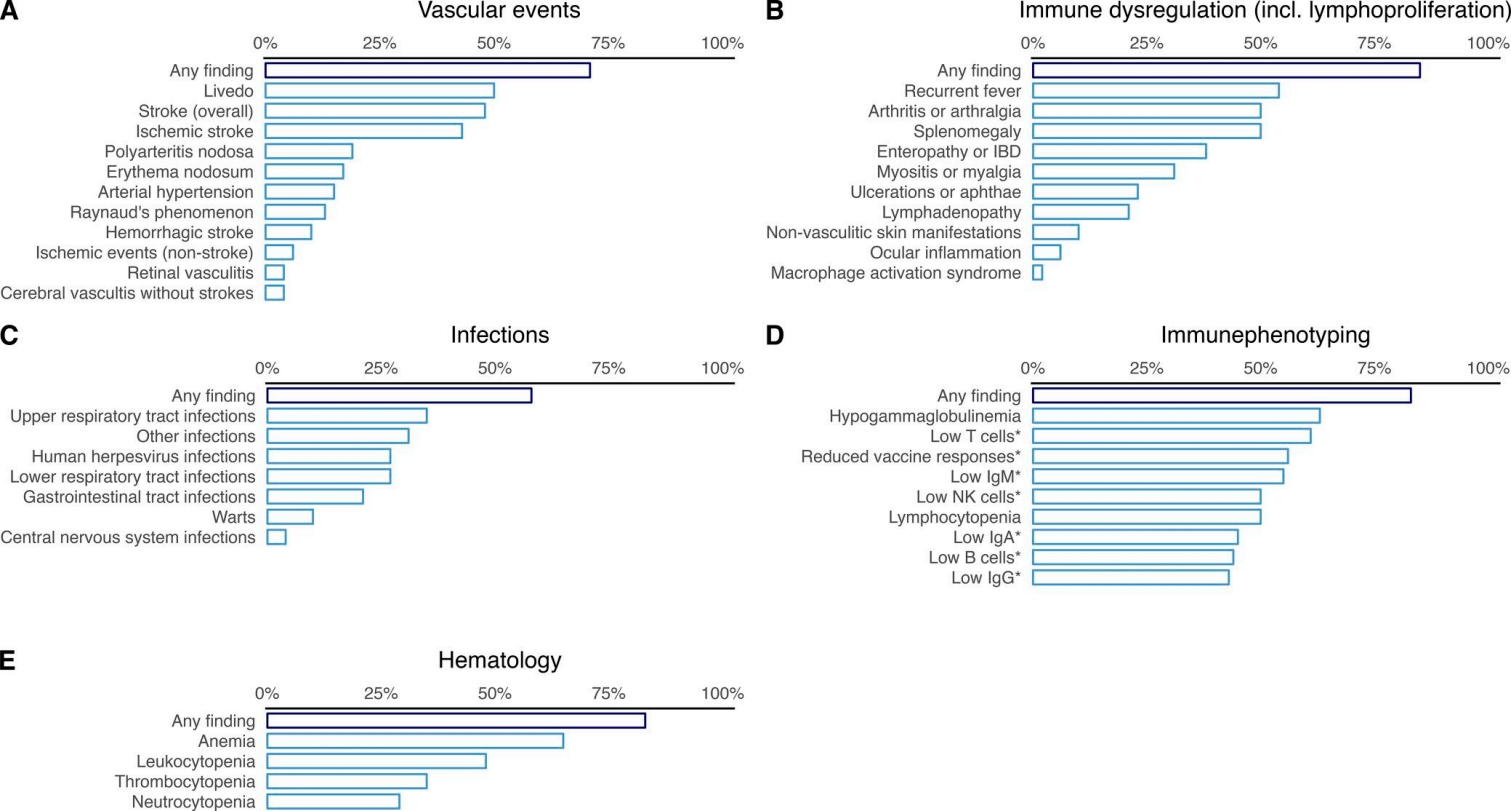
**DADA2 clinical phenotypes and laboratory immune phenotypes are heterogeneous. (A–E)** Clinical phenotypes given as fraction of a total of *n* = 48 patients. The upper bar shows the fraction of patients showing any of the clinical phenotypes displayed below. Immunophenotyping results annotated with an asterisk (*) were only reported for a subgroup of patients. Here, the fraction is calculated based on the number of patients for which a phenotyping result was reported.

In detail, strokes were documented in *n* = 23 patients (48%). Of those, *n* = 21 (43%) suffered from ischemic strokes and *n* = 5 (10%) from hemorrhagic insults. Accordingly, *n* = 3 (6%) patients were affected by both ischemic and hemorrhagic insults. Ischemic events in organs other than the central nervous system occurred in *n* = 3 (6%) patients; partially, they had various events at different locations. Of those, *n* = 2 (4%) patients had gastrointestinal ischemia, and *n* = 1 (2%) each had renal, splenic, and cardiac infarction, acral, muscle necrosis, or acute central retinal artery occlusion. The skin vasculature was affected in *n* = 32 (67%) patients. Of those, *n* = 24 (50%) had livedo racemosa (mostly of the upper and lower extremities), *n* = 9 (19%) PAN-like vasculitis (mostly affecting the skin but also the gut), *n* = 8 (17%) erythema nodosum (mostly of the lower extremities), and *n* = 6 (13%) Raynaud’s phenomenon. Additionally, *n* = 7 (15%) patients showed arterial hypertension and *n* = 2 (4%) each displayed retinal vasculitis or signs of cerebral vasculitis without evidence of strokes (Fig. 2 A).

In the group of immune dysregulation, including lymphoproliferation, recurrent bouts of fever (*n* = 26, 54%), splenomegaly (*n* = 24, 50%), and lymphadenopathy (*n* = 10, 21%), arthritis or arthralgia (*n* = 24, 50%), myositis or myalgia (*n* = 15, 32%), enteropathy or inflammatory bowel disease (IBD) (*n* = 18, 38%), ulcerations or aphthae (*n* = 11, 23%), non-vasculitis skin manifestations (*n* = 5, 10%), ocular inflammation (*n* = 3, 6%), and macrophage activation syndrome (*n* = 1, 2%) were noted (Fig. 2 B). Malnutrition or failure to thrive was documented in *n* = 4 (8%) patients, possibly related to enteropathy and/or chronic inflammation.

A high proportion of *n* = 28 (58%) patients suffered from diverse infections, as detailed in Fig. 2 C.

Abnormal laboratory findings compatible with immunodeficiency were documented in *n* = 40 (83%) patients. Of those, *n* = 24 (50%) patients had lymphocytopenia, and *n* = 30 (63%) had hypogammaglobulinemia. Immunoglobulin titers were available in *n* = 42 (88%) patients: At first consultation, IgM was low or absent in *n* = 23 (55%), IgG was low or absent in *n* = 18 (43%), and IgA was low or absent in *n* = 19 (45%). Immunoglobulin levels at last follow up did not differ significantly. Vaccination responses were assessed in *n* = 25 (52%) patients; of those, *n* = 14 (56%) had a reduced or absent response to at least one of the tested vaccines. Immunophenotyping was documented in *n* = 18 (38%) patients: At the time of diagnosis, B cell numbers were low in *n* = 8 out of 18 (44%) patients, T cell counts were low in *n* = 11 (61%), and natural killer (NK) cells were low in *n* = 9 (50%) (Fig. 2 D). At last follow-up, numbers varied in comparison to the assessment at diagnosis (Table 2). The differences between first visit and last follow-up were statistically not significant, possibly due to the small sample size. The trend toward an amelioration of low T and B cell counts may result from adequate therapy. An interferon signature was assessed in *n* = 7 (15%) patients before initiation of therapeutic immunosuppression and was elevated in all of them. In *n* = 5 patients, an interferon signature under anti-inflammatory therapy was available, showing a normalization in *n* = 4 (80%) of them (data not shown). Interferon signature after HCT was not assessed in this cohort.

**Table 2.** Immunophenotyping results

Immune cell type	Surface marker	Time point	Low	Normal	High
B cells	CD19	Diagnosis	8 (44%)	9 (50%)	1 (6%)
B cells	CD19	Last follow-up	4 (29%)	10 (71%)	0 (0%)
T cells	CD3	Diagnosis	11 (61%)	7 (39%)	0 (0%)
T cells	CD3	Last follow-up	5 (38%)	8 (62%)	0 (0%)
T helper cells	CD3 CD4	Diagnosis	6 (33%)	10 (56%)	2 (11%)
T helper cells	CD3 CD4	Last follow-up	5 (38%)	7 (54%)	1 (8%)
T killer cells	CD3 CD8	Diagnosis	10 (56%)	8 (44%)	0 (0%)
T killer cells	CD3 CD8	Last follow-up	5 (38%)	7 (54%)	1 (8%)
NK cells	CD56	Diagnosis	9 (50%)	9 (50%)	0 (0%)
NK cells	CD56	Last follow-up	3 (23%)	10 (77%)	0 (0%)

Results are given for the time point of diagnosis and at last follow-up with absolute numbers and relative percentages in parentheses. Since immunophenotyping results were not available for all patients, the percentages correspond to the total number of patients for whom a certain phenotyping result was available. Normal values were used according to Comans-Bitter et al. (17).

Hematological manifestations were found in a high proportion of patients (*n* = 40, 83%). Anemia occurred in *n* = 31 (65%) patients, leukocytopenia in *n* = 23 (48%), thrombocytopenia in *n* = 17 (35%), and neutropenia in *n* = 14 (29%) (Fig. 2 E).

### No malignancy was reported in the entire cohort

Additional organ involvement was reported in *n* = 16 (33%) patients, including kidney disease in *n* = 8 (17%), liver disease (mainly hepatomegaly) in *n* = 6 (13%), pulmonic abnormalities in *n* = 3 (6%) patients, and each *n* = 1 (2%) patient with pericardial effusion and cerebrospinal fluid pleocytosis. Neuropsychiatric symptoms were described in *n* = 11 (23%) patients. Isolated cases were reported of patients with appendicitis, transposition of large arteries, cardiomyopathy, endocrinopathy (hypothyroidism and diabetes mellitus), and coagulation disorder (data not shown).

As autoimmunity is not a hall mark feature of DADA2 and the international consensus statement of the DADA2 foundation does not recommend general autoantibody screening for DADA2 patients, we decided not to systematically assess autoimmunity. However, features of autoimmunity were reported on a case-by-case basis by some centers in three patients. Autoimmune neutropenia was reported in two patients, alongside recurrent other autoimmune cytopenias (immune thrombocytopenia, autoimmune hemolytic anemia), one of them with positive anti-neutrophil antibodies. Another patient displayed positive ANA titers and intermittently positive anti-nucleosome antibodies and lupus-anticoagulant.

### Glucocorticoids and TNF inhibitors are the conservative therapeutic mainstay and show variable efficacy

Glucocorticoid treatment was given to *n* = 34 (69%) patients. Data on treatment response were available for *n* = 29 (60%) patients. Of those, full response was achieved in *n* = 6 (21%) patients, partial response in *n* = 17 (59%), and *n* = 6 (21%) did not respond. Adverse events included infections with *n* = 2 (7%) cases of herpes zoster, and *n* = 1 (3%) each of bronchitis and submucosal dental abscess, as well as *n* = 5 (17%) cases of Cushing’s syndrome, and *n* = 1 (3%) each of steroid acne, lymphocytopenia, and hypertrichosis (data not shown).

TNF inhibition with etanercept, adalimumab, or infliximab was used in *n* = 37 (77%) patients. Of those, *n* = 30 (81%) patients received etanercept, *n* = 7 (19%) adalimumab, and *n* = 2 (5%) infliximab. Response data to TNF inhibition were available in *n* = 31 (65%) patients. Of those, full response was reported for *n* = 18 (58%) patients, partial response for *n* = 10 (32%), and no response for *n* = 3 (10%). Adverse events included *n* = 1 (3%) patient with progressive leukopenia, *n* = 1 (3%) with pruritus, and *n* = 1 (3%) with a local infection; there were no malignant complications. Among the *n* = 28 complete or partial responders, *n* = 6 (21%) reportedly discontinued. The following reasons were reported: three stopped after HCT, one died while on TNF inhibitor, one switched to a different TNF inhibitor due to an adverse event (local reaction) and subsequently had complete response, and one patient had repeatedly disrupted availability of TNF inhibitors (patient lives in Armenia). If no end date was specified, the TNF-inhibitor therapy is assumed to be ongoing. For this group of partial and complete responders, median duration of TNF-inhibitor therapy (excluding the cases that reportedly discontinued) was 2.0 years (range 0–18.5 years). Of the *n* = 13 (42%) patients with partial or no response to TNF inhibition, *n* = 5 (39%) underwent HCT (data not shown). All but one patient (*n* = 22) with stroke (hemorrhagic or ischemic) received TNF inhibitors, while only *n* = 15 of 25 (60%) of patients without stroke did.

Immunosuppressive or immunomodulatory medication other than glucocorticoids and TNF inhibitors was used in *n* = 19 (40%) DADA2 patients, including azathioprine, methotrexate, cyclophosphamide, hydroxychloroquine, mycophenolate mofetil, anakinra, nonsteroidal anti-inflammatory drugs, cyclosporine, tacrolimus, dapsone, rituximab, or daratumumab. In these cases, data on response rates were mostly incomplete (data not shown).

Immunoglobulin replacement therapy (IGRT) was given to *n* = 20 (42%) patients.

Anticoagulants were applied intermittently over short periods in *n* = 3 (6%) patients. Platelet inhibitors such as acetylsalicylic acid were used in *n* = 10 (21%) patients; of those, *n* = 5 (50%) patients were still continuing this medication at last follow-up. Other supportive medication included antihypertensive drugs, deferasirox, filgrastim, and amitriptyline as pain medication for neuropathic pain.

### HCT is feasible and effective

Overall, *n* = 10 (21%) patients received a total of 11 HCTs, of which *n* = 6 (60%) were male and *n* = 4 (40%) were female. The median age at first HCT was 6.7 years (range 2–28 years). Overall survival was good, and all patients were reported to be alive at a last follow-up. However, follow-up was short, with a median of 2 years (range 0–5 years). One patient developed secondary graft failure on day 34 after transplant due to acute graft rejection and received a successful second procedure afterward. The median time from diagnosis to HCT was short, with only 1 year (range 0–2.5 years). For details on the transplantation regimens, including donor characteristics, stem cell source, graft manipulation, conditioning regimens, and engraftment, please refer to Table S2. All three cases of acute graft-versus-host disease (GVHD) were limited to the skin. No acute GVHD of the gastrointestinal tract or the liver was reported, and all cases of acute GVHD were mild overall (overall grade I in *n* = 2, and II in *n* = 1). Also, no chronic GVHD was reported. Infectious complications occurred in *n* = 6 (60%) patients. After HCT, residual disease symptoms were noted in *n* = 2 (20%) patients; *n* = 1 (10%) had Raynaud’s phenomenon, and *n* = 1 (10%) displayed transient autoinflammation, both had full donor chimerism at last follow-up. Importantly, no ischemic events were reported as relapsing symptoms following HCT, while *n* = 4 of 10 HCT patients (40%) had strokes before transplant. Donor chimerism was reported as a global measurement for *n* = 9 (90%) patients, while for *n* = 1 patient (10%) only lineage-specific chimerism was reported. Global chimerism was near complete (i.e., 90–95%) in *n* = 2 (20%) and complete (i.e., > 95%) in *n* = 7 (70%), including in the patient undergoing a second procedure due to graft failure. In the remaining case, only lineage-specific chimerism was reported. In this patient, donor chimerism was low in T cells (34%), while being near complete in NK cells and complete in monocytes and B cells.

### Earlier disease onset of DADA2 correlates with the likelihood of HCT and the development of IBD, while hypogammaglobulinemia worsens with age

**Figure 3.**
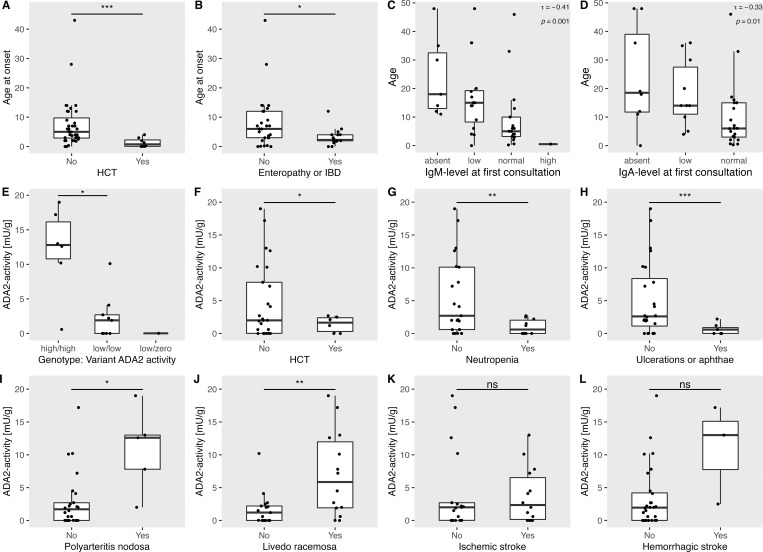
**DADA2 phenotypes follow distinct demographic, clinical, therapeutic, genetic, and biochemical correlations.** Relation of clinical phenotype, age at disease onset, age, ex vivo ADA2 enzymatic activity, and allele activity in vitro. Boxplots show median, 25th, and 75th percentile, and whiskers with minimum/maximum value (or 1.5 × interquartile range with outliers). Significance levels based on Welch’s *t* test. **(A and B)** Age of disease onset depending on whether patients underwent HCT or suffered from enteropathy or IBD. **(C and D)** Levels of IgM and IgA at first consultation in relation to age. **(E)** Patient ADA2 activity (ex vivo) in relation to the variant ADA2 activity (determined in vitro). **(F)** Patient ADA2 activity in patients that underwent HCT and those that did not. **(G–L)** Patient ADA2 activity depending on the presence of certain disease phenotypes. *P < 0.05, **P < 0.01, ***P < 0.001.

For *n* = 8 (80%) patients who received HCT, the age of onset was reported. Disease onset was documented within the first year of life in *n* = 4 (50%) patients, and all had disease onset before the age of 5 years. Patients who received HCT had a significantly lower age of disease onset than those who did not (1.3 vs. 7.5 years, P = 0.0002). This was also true when using a logistic regression model (P = 0.0003, Fig. 3 A).

In addition, patients suffering from enteropathy or IBD showed a significantly lower age of disease onset (3.2 vs. 8.7 years, P = 0.014). Again, this finding was robust using a logistic regression model (P = 0.010, Fig. 3 B).

Finally, hypogammaglobulinemia was more pronounced with increasing age. Levels of IgM, IgG, and IgA were reported in categorical levels (“absent,” “low,” “normal,” and “increased”). Analysis was done only for IgM and IgA levels, as many patients received IGRT, distorting the analysis for IgG. At first consultation, data on IgM and IgA levels, including corresponding age were available in *n* = 41 (85%) and *n* = 40 (83%) patients, respectively. Using Kendall’s rank correlation, a significant negative correlation of age at first consultation with levels of IgM and IgA (tau = −0.408, P = 0.0010 and tau = −0.3295605, P = 0.0096, respectively) was established (Fig. 3, C and D).

The remaining clinical symptoms did not show significant association with the age of disease onset.

### Variant ADA2 activity correlates with patient ADA2 activity

**Figure 4.**
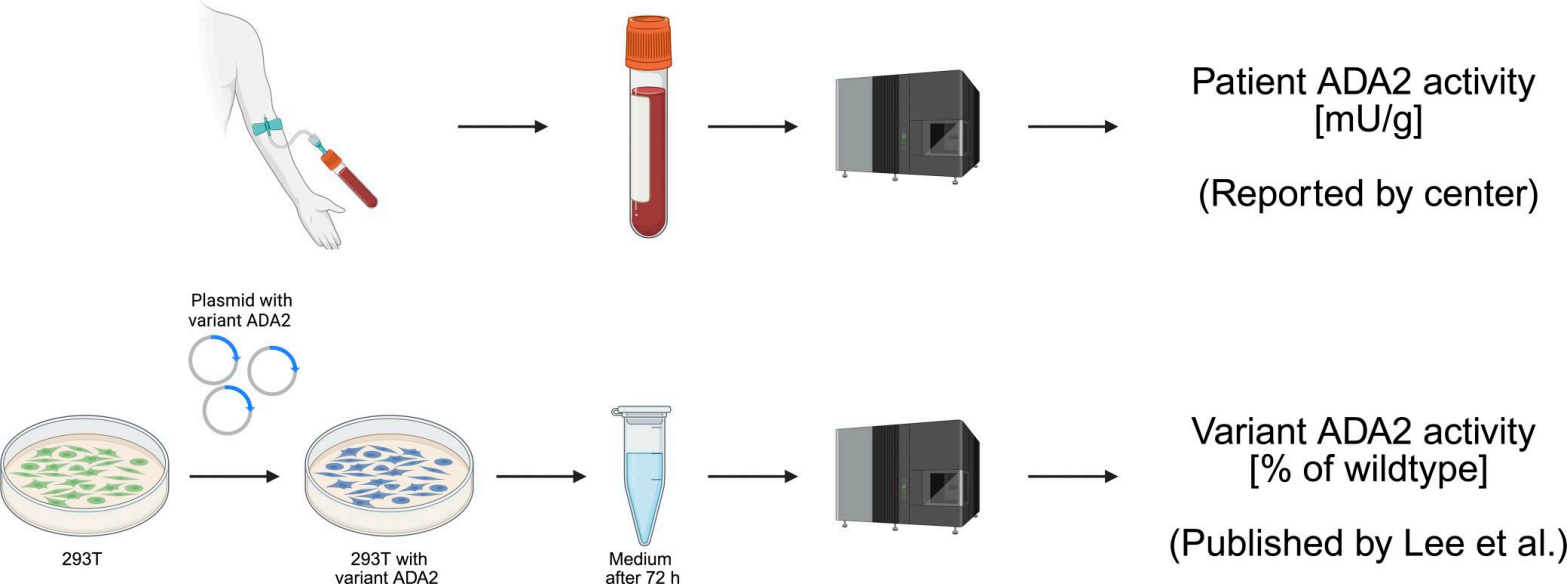
**Residual ADA2 activity of patients and variants are distinct properties.** Above: Patient ADA2 activity is measured in blood samples ex vivo. Values were reported by the respective centers, commonly in mU/g. Below: The ADA2 activity of a specific genetic variant can be determined in vitro by expressing the variant in question in a cell line. ADA2 activity is then measured from the supernatant medium and reported as percentage of wild-type activity. Lee et al. established this for a number of common ADA2 variants, and classified the residual ADA2 activity as high (>3%), low (<3%), or zero (13). Our analysis is based on the published values.

**Figure 5.**
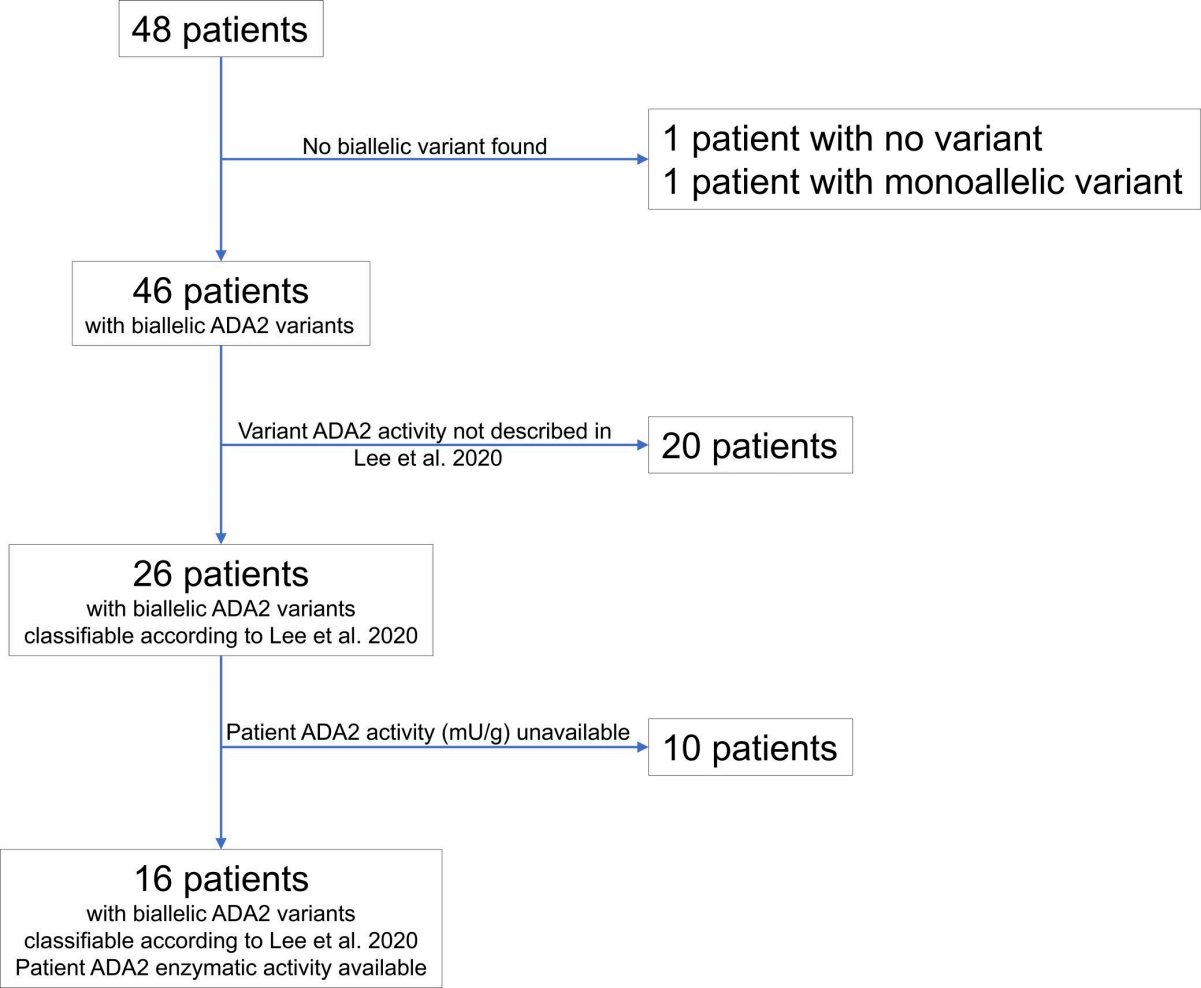
**Classification algorithm for the correlation of patient and variant ADA2 activity.** Allele classification according to Lee et al. (13) and correlation to patients’ (ex vivo) ADA2 enzymatic activity. The 16 final patients could be analyzed for correlation of their (ex vivo) ADA2 activity and the ADA2 activity of their genetic variants (13).

To determine whether *ADA2* alleles and the variant or patient ADA2 activity correlate with DADA2 phenotype, we applied the categorization coined by Lee et al. (13), which predicts a variant’s residual ADA2 activity based on in vitro measurement (Fig. 4). Based on this prediction, an allele is assigned into one of the following groups: High residual variant activity (>3% of wild-type activity), low residual variant activity (<3% of wild-type activity), and no residual variant activity (0% of wildtype activity). A total of 17 out of 33 *ADA2* variant alleles in our cohort could be classified accordingly (Fig. 1 and Table S1). For *n* = 26 (54%) patients, both *ADA2* alleles could be categorized in this manner. Out of these, patient ADA2 activity (measured in mU/g) was available for *n* = 16, allowing for correlation of the variant ADA2 activity and patient ADA2 activity. For an overview of the classification algorithm used to assess the correlation between patient and variant ADA2 activity, please see Fig. 5. Of those, *n* = 6 patients carried two *ADA2* variants with high ADA2 activity, (“high/high”), *n* = 9 patients carried two variants with low ADA2 activity (“low/low”), and *n* = 1 patient carried one variant with low and one variant with zero ADA2 activity (“low/zero”). There was a significant difference between the high/high and the low/low group in patient ADA2 activity (mean 12.1 vs. 2.3 mU/g, P = 0.01, Fig. 3 E), suggesting that in this subgroup, the variant ADA2 activity published by Lee et al. correctly predicts patient ADA2 activity. Since there was only *n* = 1 patient with a low/zero allele combination, statistical analysis with this subgroup was not possible; however, the reported plasma ADA2 activity for this patient was 0 mU/g.

### Lower variant ADA2 activity is associated with HCT decision

**Figure S2.**
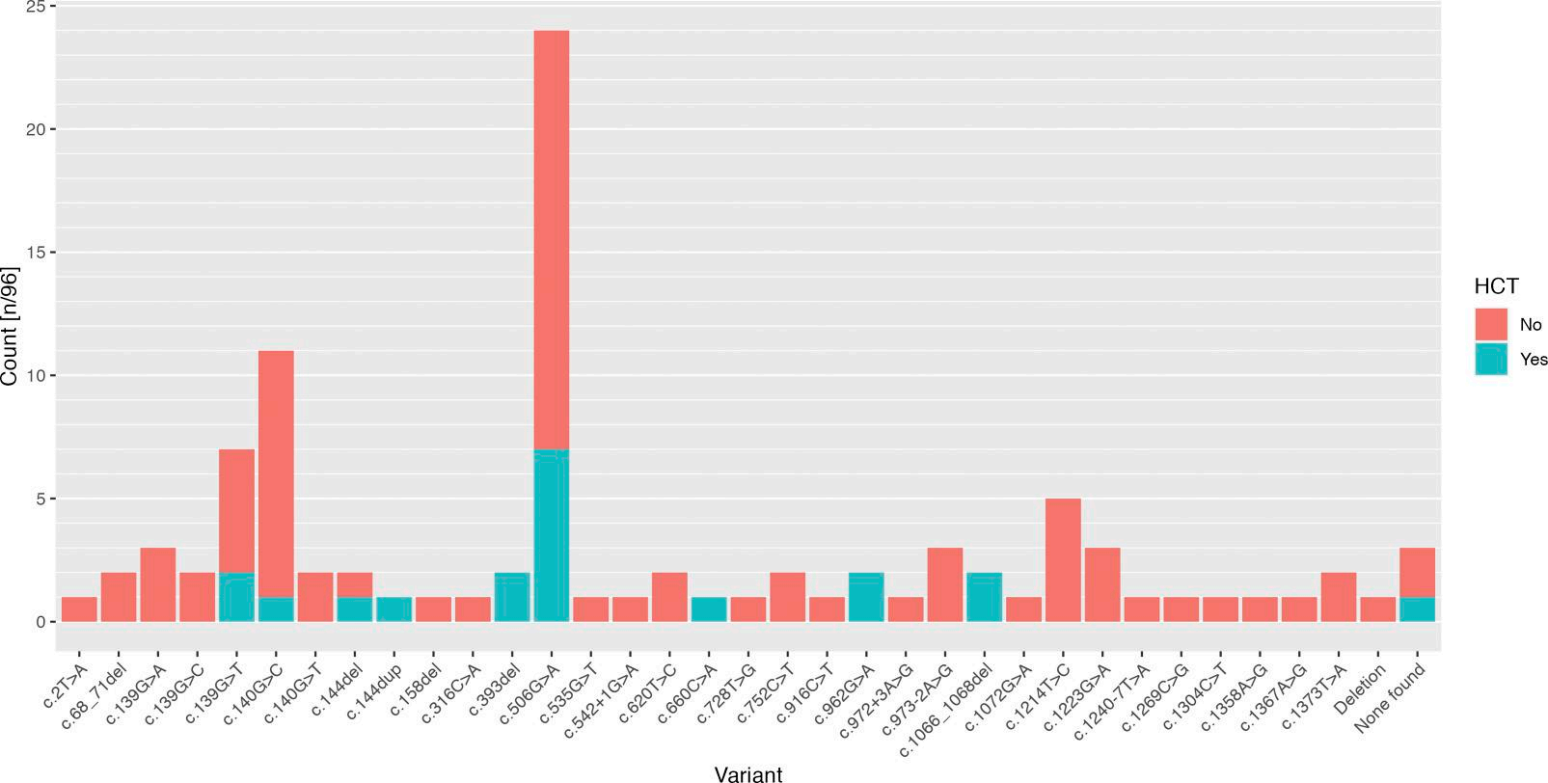
**A histogram of all variants found in this cohort.** If the carrier underwent HCT, the variant was colored blue; if not, it was colored red.

In the group of 10 patients treated with HCT, 10 unique *ADA2* variants accounted for all 20 alleles. 14 of these 20 alleles, corresponding to 7 of the 10 distinct variants, could be characterized as described above (Fig. 4, Fig. 5, Fig. S2, and Table S1). Remarkably, in the HCT group, only 1 of these 14 variant alleles (7%) showed high ADA2 activity, in contrast to 18 out of 50 (36%) in the non-HCT group (Table S3). Using Kendall’s rank correlation, a significant negative correlation (tau = −0.28, P = 0.021) between the category of each allele (high, low, and zero variant activity) and the probability of the carrier receiving HCT was found. In contrast, when looking at the unique *ADA2* variants only, no clear genotype–phenotype correlation could be established (Fig. S2).

### Low patient ADA2 activity correlates with neutropenia, while high patient ADA2 activity correlates with vasculitis symptoms

To analyze whether patient ADA2 activity was correlated to a certain clinical phenotype, we compared their ADA2 activity to clinical characteristics. In this analysis, all 31 patients with ADA2 activity measured in mU/g were included.

When looking at patients who underwent HCT, there was a trend toward lower patient ADA2 enzymatic activity (1.4 vs. 4.8 mU/g, P = 0.01, Fig. 3 F). However, while this finding was statistically significant when using Welch’s *t* test, statistical significance was not upheld when using a logistic regression model (P = 0.09) or Wilcoxon rank-sum test (P = 0.33). The significant result observed with Welch’s *t* test is likely attributable to a few patients with comparably high residual activity not receiving HCT. Most likely the study was also insufficiently powered to answer this question, as patient ADA2 activity was only available in 6 out of 10 patients receiving HCT.

We found a significantly lower patient ADA2 activity in individuals with neutropenia compared to those with normal neutrophil counts (1.0 vs. 5.3 mU/g, P = 0.005). Patient ADA2 activity was also lower in individuals suffering from aphthae or ulcerations (0.7 vs. 5.1 mU/g, P = 0.001), possibly as a clinical surrogate of neutropenia. On the other hand, patients with vasculitis symptoms such as livedo racemosa or PAN-like vasculitis had significantly higher ADA2 activity (7.0 vs. 1.7 mU/g, P = 0.01, and 10.9 vs. 2.8 mU/g, P = 0.04, respectively) (Fig 3, G–J). All these findings were also statistically significant when using a logistic regression model or a Wilcoxon rank-sum test. However, there were no significant differences in ADA2 activity between patients with and without ischemic or hemorrhagic stroke (Fig. 3, K and L). Also, there were no significant differences in patients with infections (data not shown). Of note, we could not find a significant correlation between age of onset and levels of patient ADA2 activity either (P = 0.16), and there was no significant correlation between patient ADA2 activity with regard to response to TNF inhibition (data not shown).

## Discussion

According to the International Consensus Statement of the DADA2 Consensus Committee 2023 (18), four different phenotypes of DADA2 can be defined: inflammatory and/or vasculitic, hematologic, immunodeficient, and presymptomatic. When comparing the distribution of these main features in our cohort with the current biomedical literature (10, 19, 20), we found a higher proportion of patients with hematological features (83%). Also, features of immunodeficiency were more prominent (85%). Coherently, immunophenotyping also revealed a higher proportion of abnormalities (83%), and there were more infectious diseases (85%). Overall, these phenomena are most likely due to differences in recruitment strategies underlying the distinct cohorts. It is possible that this cohort is skewed toward patients with immunodeficiency and hematological manifestations. However, it may also be due to the increasing awareness of these manifestations of DADA2 by physician-scientists not primarily involved in inborn errors of immunity and, consequently, by previous underreporting.

There is an international consensus on TNF inhibitors as gold-standard care for the vasculitic features of DADA2 (18). However, response rates for TNF inhibitors differ. In a literature review including 131 patients with TNF inhibitor treatment, they were effective in 78% (21). In our cohort, TNF inhibitors showed full response in merely 58%, possibly due to a skewing of the cohort toward patients with hematological and immunological symptoms. This is in line with reports showing that hematological involvement and recurrent infections are associated with poor effectiveness of TNF inhibitors (13, 18, 21, 22). As expected, disease control with steroids was insufficient, as were other forms of immunosuppression, adding support for the international consensus of TNF inhibitors as first-line anti-inflammatory therapy for the vasculitic features of DADA2 (18).

The possible skewing of this cohort toward patients with a predominantly hematological phenotype may explain the high proportion of individuals undergoing HCT. However, an increasing willingness to perform HCT in this disease due to favorable outcome data (14) and early disease diagnosis may also be a contributing factor. HCT outcome was favorable in all our patients. In this cohort, patients who received HCT had a significantly lower age of disease onset. This finding is likely due to a greater readiness to consider HCT in younger children because of a more favorable risk profile. However, it may also be a sign of greater disease severity in patients with early onset of DADA2 (23). Yet, this study did not systematically assess disease severity, and there were no other significant correlations of disease onset and clinical phenotype that indirectly suggest such a connection.

To our knowledge, this is the first study that showed that variant ADA2 activity (13) correctly predicts patient ADA2 activity. Intriguingly, we found a significantly lower patient ADA2 activity in individuals with neutropenia. Fittingly, this was also true for patients with aphthae or ulcerations—a typical symptom of neutropenia, even though other inflammatory causes for these symptoms cannot be ruled out. On the contrary, in cases with livedo racemosa or PAN-like vasculitis, we observed significantly higher patient ADA2 activity. PAN-like vasculitis and livedo racemosa are commonly considered part of the vasculitis spectrum. On the other hand, neutropenia may indicate bone marrow failure. Fittingly, carrying variants with lower residual ADA2 activity was also correlated to HCT in our cohort. Lee et al. reported that, among DADA2 patients, lower variant ADA2 activity may predispose to bone marrow failure, while higher variant ADA2 activity confers the “classical” vasculitis phenotype (13). Moreover, Andriessen et al. (24) observed a trend toward higher residual ADA2 activity in patients with vasculitis compared to those with a hematologic phenotype. Our study was able to confirm this observation, reaching statistical significance. Thereby, we add a substantial piece of evidence to the picture that was previously mainly based on in vitro data.

It is important to note that our study did not find a significant association between patient ADA2 activity and stroke—the defining vasculitis feature of this disease. Also, it needs to be considered that neutropenia, HCT, age of disease onset, and possibly genotype are not necessarily independent variables, but rather interrelated features of the severe hematologic manifestations of DADA2. This needs to be taken into account when interpreting the fact that ADA2 activity correlates with these features. Specifically, the observed correlation between low variant ADA2 activity and HCT may be mediated through a certain clinical phenotype, possibly bone marrow failure.

In general, it should be emphasized that in our study, the correlations between variant and patient ADA2 activity could only be analyzed in a subgroup of 16 patients, and the correlations of patient ADA2 activity and clinical phenotype in a subgroup of 31 patients, as both patient ADA2 activity was not uniformly reported, and variant activity (13) was not available for all genotypes observed in this cohort. With DADA2 being a rare disease, powerful statistical analysis is inherently constrained by low case numbers. Additionally, as this is a retrospective study, data on patient ADA2 activity relied on information provided by the individual centers. A centralized evaluation of patient samples was not within the scope of this study, potentially introducing assay-dependent variability. Furthermore, because our dataset included only a single measurement per patient, the extent of intra-individual variation could not be assessed. To our knowledge, most ADA2 activity assays have been optimized to distinguish healthy individuals from those with DADA2 and may lack the necessary precision to reliably assess differences in ADA2 activity at very low levels. Determining metrics of an assay designed for this specific question, such as lower limit of detection, the basis of determining the cutoff and diagnostic range for DADA2, as well as the coefficient of variation for measurement of DADA2 samples will be important in future prospective and experimental studies. This is essential to provide the global community with access to a validated methodology, as both prognosis and treatment decisions might depend on it.

Moreover, genotype and residual ADA2 activity are surely not the only factors determining disease severity, given the clinical heterogeneity of DADA2, incomplete penetrance, and variable manifestations of identical *ADA2* genotypes. Mechanistical understanding of how specific variants affect the function of ADA2 has just started to emerge (11). Probably, other factors like epigenetics, genetic modifiers, and patient environment also play an important role in the manifestation of DADA2. To address these questions and to confirm the correlations observed in our study, further studies and larger international cohorts are essential, showing the need for the international patient registry of this rare disease, which is currently being built (25).

In summary, prediction of clinical phenotype or disease severity based on genotype and patient ADA2 activity remains limited but may become a contributing factor in evaluating this multifaceted disease. Despite the limitations of our study explained above, the coherent and significant correlations observed in our cohort support the validity of our findings and strongly encourage further research on residual ADA2 activity across patients with different DADA2 phenotypes, as well as a comprehensive classification of ADA2 variants based on their activity in vitro, as initiated by Lee et al. Following further evaluation, the observed correlations may inform treatment strategies, particularly in presymptomatic patients, patients with rare phenotypes, or patients irresponsive to TNF inhibitors.

To our knowledge, this is the first study that provides primary patient data indicating that variant ADA2 activity (13) correctly predicts patient ADA2 activity. Additionally, we find clinical evidence supporting the hypothesis that patients with low plasma ADA2 activity are prone to bone marrow failure, while patients with higher plasma ADA2 activity may rather present with vasculitis symptoms. We also observe that genotypes comprising low-activity ADA2 variants are associated with an increased likelihood of undergoing HCT. Further clinical and experimental research is warranted to corroborate these correlations and, in the future, may even help to guide treatment decisions.

## Materials and methods

The multicenter retrospective study was conducted in accordance with the ethical standards of the responsible committee on human experimentation (institutional and national) and the Helsinki Declaration of 1975, as revised in 2013, and was approved by the ethical review board of the Ludwig-Maximilian University of Munich (Institutional Review Board project number 21-0274).

Inclusion criteria were deficiency of ADA2, diagnosed either by genetic testing showing biallelic pathogenic variants in *ADA2* or significantly decreased ADA2 activity (>2 standard deviations below the median) in whole blood plasma. A patient’s clinical features were considered as core data. Therefore, the completion of the clinical phenotype questionnaire was required to include a submission. We excluded incomplete submissions and duplicates. Data were submitted from collaborating hospitals in Germany, Austria, and Switzerland after open calls via the “Arbeitsgemeinschaft Pädiatrische Immunologie” and the “Gesellschaft für Pädiatrische Onkologie und Hämatologie.”

Data collection was conducted via the online survey tool LimeSurvey from April 2021 until June 2022. Data were entered by representatives of the distinct centers; therefore, the reporting of certain symptoms and responses to treatment was based on the treating physician’s assessment. Data analysis was done using RStudio IDE (Posit PBC, Version 1.3.1073 for macOS).

ADA2 plasma activity had been determined upon primary diagnosis and was reported retrospectively by the treating physicians of the DADA2 patients when available. No new blood samples were taken for the purpose of this retrospective study. In the majority of cases, ADA2 activity had been measured in extracts of dried plasma spots. These 31 patient samples were measured in the same laboratory (Purine Metabolic and Immunodeficiency Lab, Duke University Medical Center, Department: Medicine) by normalizing ADA2 activity to grams of extract protein (mU/g) using an high pressure liquid chromatography (HPLC) assay as described previously (26, 27, 28). For every patient, a single measurement was reported by the respective centers. All statistical analysis on patient ADA2 activity was based on this subgroup of 31 patients. In four additional DADA2 patients, plasma ADA2 activity was reported as mU/ml. For these samples, neither details on the assay used nor normal values were available; therefore, we did not include them in statistical analyses.

For statistical inference, Welch’s *t* test, Wilcoxon rank-sum test, ANOVA, likelihood-ratio test, and Kendall’s τ coefficient were used. When not explicitly stated otherwise, P values given for intergroup comparisons of continuous data were determined by Welch’s *t* test. P values for logistic regression models involving continuous predictors and a binary outcome variable were calculated by using the likelihood-ratio test. In all other cases, the statistical test is stated in the accompanying figure legend. Due to the exploratory nature of this study, no adjustment for multiple testing was performed.

Fig. 4 and Fig. S1 were generated using the platform BioRender (biorender.com).

### Online Supplemental material

The supplementary material includes two figures and three tables. Fig. S1 gives a visualization of symptoms of DADA2 and the observed frequencies in this cohort. Fig. S2 displays the different variant alleles found in this cohort and whether or not the carriers underwent HCT. Table S1 provides details on the variant alleles, such as allele frequency, prediction scores, type of mutation, and the residual ADA2 activity described by Lee et al. (13). Details on patients that underwent HCT are given in Table S2. Table S3 shows the correlation variant ADA2 activity and whether the carrier underwent HCT.

## Supplementary Material

Table S1 shows overview of variants.

Table S2 shows data of patients that underwent HCT.

Table S3 shows the correlation variant ADA2 activity and whether the carrier underwent HCT.

## Data Availability

The data underlying this study are not publicly available to protect patient privacy. The data are available from the corresponding author upon reasonable request.
